# Insulin-Like Peptides and the Target of Rapamycin Pathway Coordinately Regulate Blood Digestion and Egg Maturation in the Mosquito *Aedes aegypti*

**DOI:** 10.1371/journal.pone.0020401

**Published:** 2011-05-27

**Authors:** Monika Gulia-Nuss, Anne E. Robertson, Mark R. Brown, Michael R. Strand

**Affiliations:** 1 Department of Entomology, University of Georgia, Athens, Georgia, United States of America; 2 Center for Tropical and Emerging Global Diseases, University of Georgia, Athens, Georgia, United States of America; New Mexico State University, United States of America

## Abstract

**Background:**

Mosquitoes are insects that vector many serious pathogens to humans and other vertebrates. Most mosquitoes must feed on the blood of a vertebrate host to produce eggs. In turn, multiple cycles of blood feeding promote frequent contacts with hosts and make mosquitoes ideal disease vectors. Both hormonal and nutritional factors are involved in regulating egg development in the mosquito, *Aedes aegypti*. However, the processes that regulate digestion of the blood meal remain unclear.

**Methodology/Principal Findings:**

Here we report that insulin peptide 3 (ILP3) directly stimulated late phase trypsin-like gene expression in blood fed females. *In vivo* knockdown of the mosquito insulin receptor (MIR) by RNA interference (RNAi) delayed but did not fully inhibit trypsin-like gene expression in the midgut, ecdysteroid (ECD) production by ovaries, and vitellogenin (Vg) expression by the fat body. In contrast, *in vivo* treatment with double-stranded MIR RNA and rapamycin completely blocked egg production. *In vitro* experiments showed that amino acids did not simulate late phase trypsin-like gene expression in the midgut or ECD production by the ovaries. However, amino acids did enhance ILP3-mediated stimulation of trypsin-like gene expression and ECD production.

**Conclusions/Significance:**

Overall, our results indicate that ILPs from the brain synchronize blood meal digestion and amino acid availability with ovarian ECD production to maximize Vg expression by the fat body. The activation of digestion by ILPs may also underlie the growth promoting effects of insulin and TOR signaling in other species.

## Introduction

Most female mosquitoes feed on the blood of vertebrates to produce eggs. Multiple cycles of blood feeding and egg development promote frequent contacts with hosts, making mosquitoes ideal vectors for transmission of several disease-causing organisms to humans and other mammals. For the yellow fever mosquito, *Aedes aegypti*, each reproductive cycle begins with the ingestion of blood, which is digested in the midgut into amino acids that are used by the fat body for the biosynthesis of yolk proteins (YPs) [1]. YPs are then packaged into oocytes that greatly increase in size followed by chorion formation and oviposition between 72 and 96 h post bloodmeal (pbm).

Regulation of these events in *A. aegypti* involves both hormonal and nutritional cues (reviewed in [2]). Within 1 h of blood feeding, neurosecretory cells in the brain release insulin-like peptides (ILPs) and other neuroendocrine factors, which stimulate the ovaries to secrete ecdysteroid hormones (ECDs) into the hemolymph up to 30 h pbm [3], [4]. This process is regarded as a priming step for egg maturation, because of the involvement of ECD signaling in activation of vitellogenesis ([2], [5], see below). Concurrently, entry of the blood meal into the midgut results in the biphasic release of trypsin-like enzymes with an early trypsin-like protease (AaET) detected at 3 h pbm and the inducible expression of multiple late phase trypsin-like proteases occurring between 12 and 30 h pbm [6]–[10].

Late phase trypsin-like activity is responsible for the majority of blood meal digestion: releasing approximately two thirds of available amino acids, which after absorption are transported through the circulatory system to other tissues [1], [8], [9], [11]. In addition to being used by the fat body for biosynthesis of YPs, amino acid sensing through the target of rapamycin (TOR) pathway also elevates transcription of the major YP, vitellogenin *Vg* [5], [12], [13], [14]. The ECD, 20-OH ecdysone (20E), and exogenous insulin enhance amino acid dependent activation of *Vg* expression in isolated fat body, whereas inhibition of target of rapamycin (TOR) signaling by the inhibitor rapamycin reduces *Vg* expression [5]. At least two (ILP3 and ILP4) of the eight ILPs encoded by *A. aegypti* activate the insulin signaling pathway [3], [4]. ILP3 binds to the mosquito insulin receptor (MIR) with high affinity, while ILP4 binds to another membrane protein, which appears to interact with the MIR. Both ILPs are expressed in brain neurosecretory cells, and decapitation of females immediately after blood feeding prevents egg maturation [3], [15]. Reciprocally, injection of a single dose of either ILP3 or ILP4 restores vitellogenesis in decapitated females as judged by yolk deposition in oocytes.

Amino acid sensing through TOR is also implicated in regulating the translation and secretion of AaET by the midgut [16]. The factors regulating expression of late phase trypsin-like genes in contrast remain unclear although decapitation of mosquitoes within 1 h of blood feeding does reduce late phase midgut trypsin activity [17]. Here we report that insulin and TOR signaling interact to regulate late phase trypsin-like gene expression and blood meal digestion in *A. aegypti*. Our results also suggest that the well-established growth promoting effects of insulin in model animals could involve a previously unrecognized role for the insulin signaling pathway in regulating digestion.

## Results

### 2.1. ILP3 restores late-phase midgut trypsin activity in blood-fed, decapitated females

**Figure 1:**
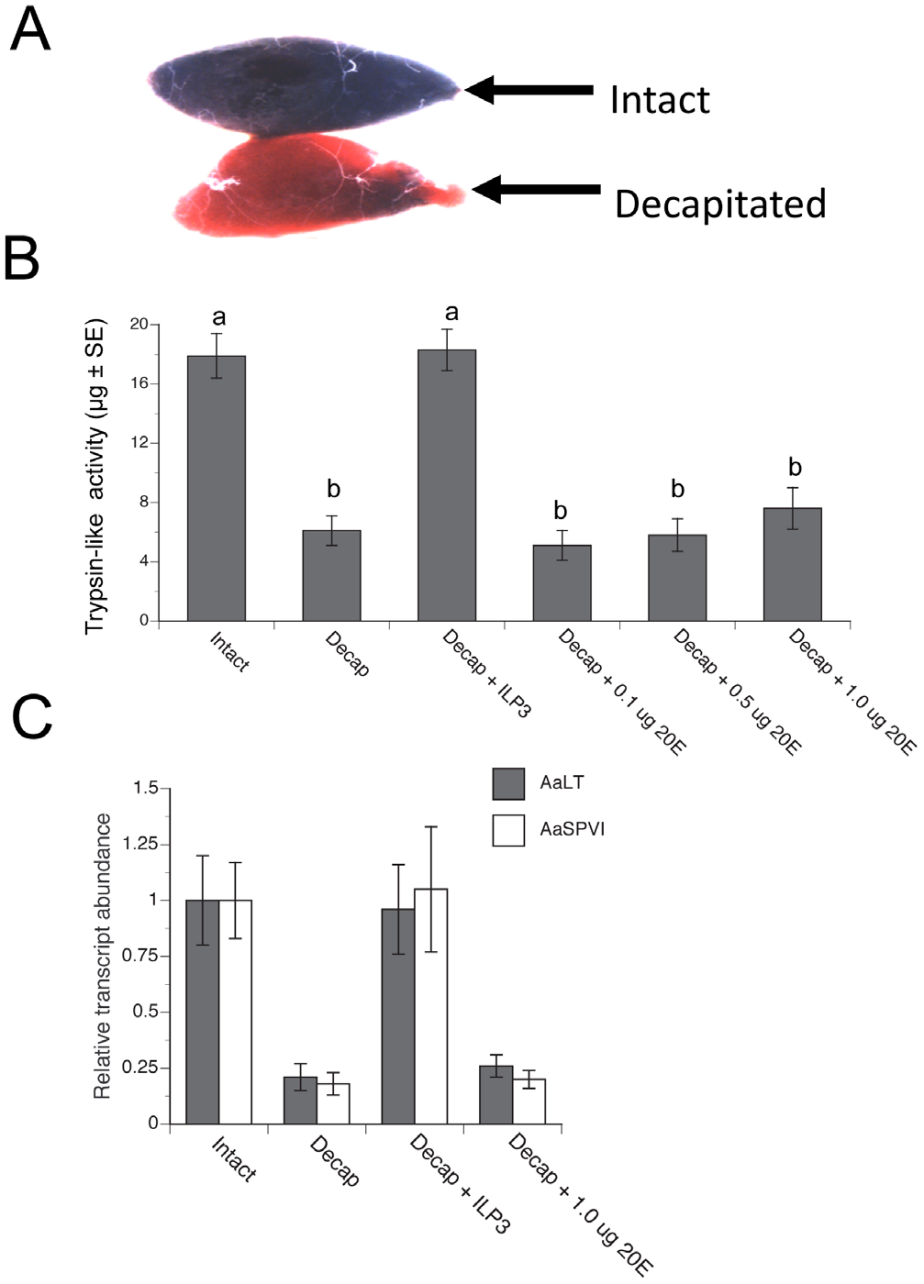
ILP3 rescues midgut trypsin activity and late trypsin expression in blood-fed, decapitated females. (A) Light micrograph of midguts dissected from an intact and decapitated female at 16 h post blood meal (pbm). The dark red color of the blood bolus from the intact female indicates normal digestion, whereas the bright red color of the blood bolus from a decapitated female indicates delayed digestion. (B) Trypsin-like activity at 24 h pbm in the midgut of blood-fed intact females (positive control) compared to blood-fed, decapitated females injected with saline (Decap), 20 pmol of ILP3 (Decap + ILP3), or 0.1 µg–1 µg of 20-hydroxyecdysone (20E) (Decap + 20E). Different letters above a given treatment indicate means that significantly differ from the intact positive control (F_5, 83_ = 14.0, P<0.001; followed by comparison of means to the control using Dunnett's multiple comparison procedure, α = 0.05). (C) rqRT-PCR analysis of *AaLT* and *AaSPVI* expression in midguts at 24 h pbm from intact females and decapitated females injected with saline, 20 pmol ILP3, or 1 µg 20E. Transcript levels are standardized to a level of 1 for midguts from intact females, while transcript levels for other treatments are expressed relative to the intact midgut control. Each treatment was replicated four times using samples of midguts collected from four females.

Blood meal digestion visually correlates with the bolus changing from a bright red to a dark, near black color by 12 h pbm followed by a progressive decline in size before excretion between 32 to 40 h pbm (Fig. 1A). In the current study, we observed that decapitation within 1 h pbm delayed the color change of the blood bolus from 12 h to approximately 36 h pbm (Fig. 1A). Consistent with this observation and prior results [17], late phase trypsin-like activity at 24 h pbm was 68% lower in midguts from decapitated females than in midguts from intact females (non-decapitated, control) (Fig. 1B). Given that a single dose of ILP3 (20 pmol) rescues egg maturation in blood-fed decapitated females [3], we asked whether the same dose of ILP3 rescued midgut trypsin-like activity in decapitated females. Our results showed that 20 pmol of ILP3 restored midgut trypsin activity to control levels at 24 h pbm (Fig. 1B). In contrast, injection of 20E over a range of concentrations that stimulate *Vg* transcription in fat bodies *in vitro* [12] had no rescue effect on midgut trypsin-like activity (Fig. 1B).

*Ae. aegypti* expresses 12 serine protease (SP)-like genes in the midgut [6], [8]. Transcription of early trypsin (*AaET*) occurs prior to blood feeding while serine protease-like genes II–V (*AaSPII*–*V*) are constitutively expressed [6], [9]. In contrast, the SP gene originally named late trypsin (*AaLT*) as well as *AaSPI*, *AaSPVI (5G1)*, and *AaSPVII* are each inducibly expressed in the midgut between 12 and 24 h pbm [6]. Functional studies implicate *AaLT, AaSPVI* and *AaSPVII* in blood meal digestion and reduced egg development [8]. However, *AaSPVI* accounts for most late phase trypsin-like activity, while also exhibiting structural features and substrate usage activity consistent with being a classic trypsin enzyme [6], [8]. To assess whether decapitation at 1 h pbm blocked the inducible expression of late phase serine protease-like genes, we measured transcript abundance of *AaSPVI* and *AaLT* by rqRT-PCR. Our results showed that the abundance of each was much lower in decapitated individuals at 24 h pbm than in intact controls (Fig. 1C). Moreover, injection of ILP3 into decapitated females fully restored expression levels of *AaSPVI* and *AaLT* to control levels while 20E did not (Fig. 1C).

### 2.2. *In vivo* knockdown of the MIR delays blood meal digestion

**Figure 2:**
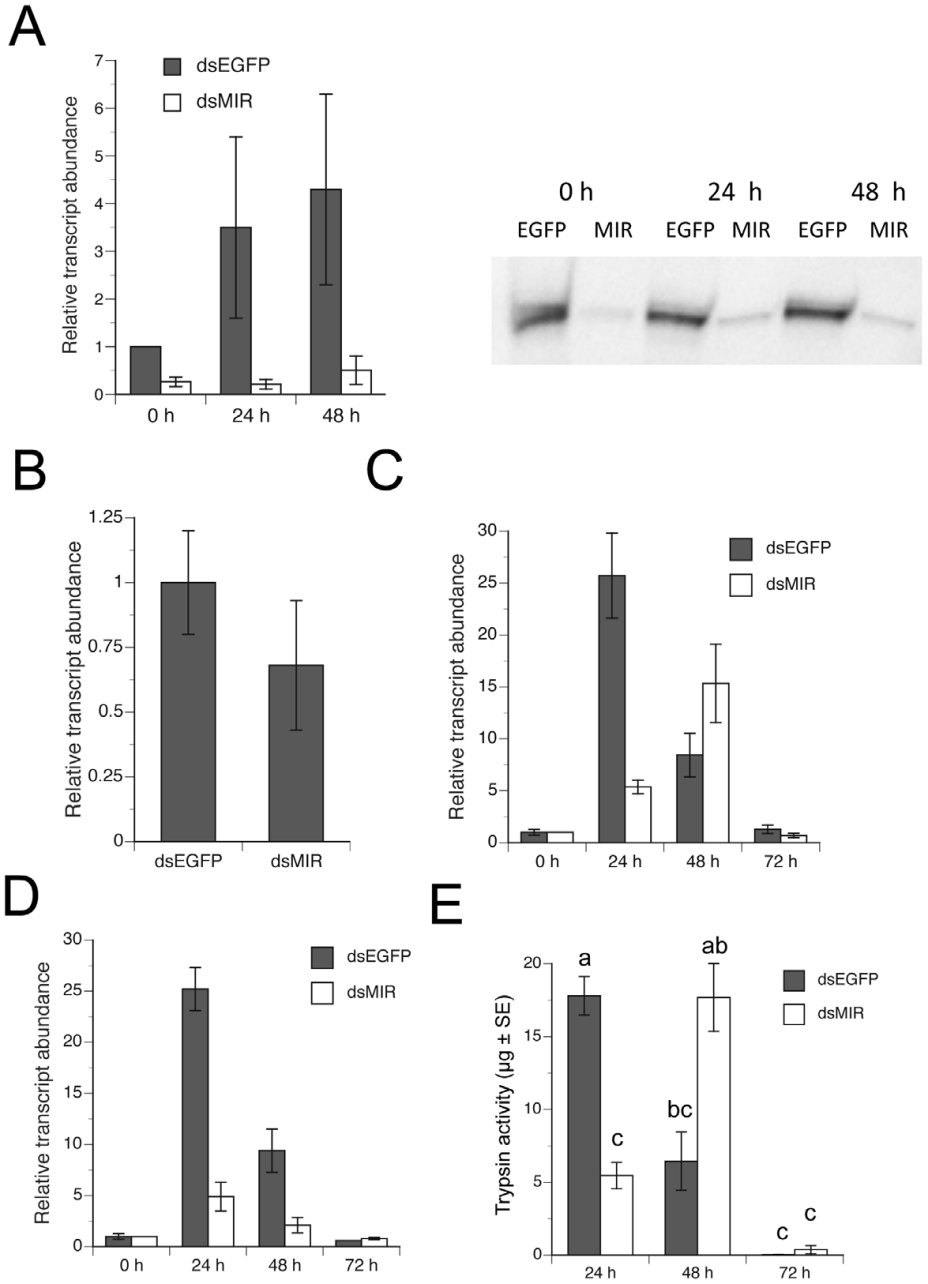
Knockdown of MIR expression in the midgut of females delays late trypsin expression and activity after blood ingestion. (A) Left panel: rqRT-PCR analysis of *MIR* transcript abundance in mosquito midguts 0–48 h pbm from females (blood-fed 6 days old) treated with *dsEGFP* or *dsMIR* RNA at 1 day post eclosion. Transcript levels were standardized as described in Fig. 1C with each treatment replicated four times using samples of midguts collected from four females. Right panel: The immunoblot shows MIR expression in midguts (4 midguts per lane) collected 0–48 h pbm from females treated as above after SDS-PAGE, transfer to nitrocellulose, and detection with an MIR antibody. (B) rqRT-PCR analysis of *AaET* transcript abundance in midguts 2 h pbm from females treated with *dsEGFP* or *dsMIR* RNA. Transcript levels were standardized to 1 for *dsEGFP*-treated females (negative control) while transcript abundance for *dsMIR*-treated females is expressed relative to the negative control. Each treatment was replicated three times using samples of midguts collected from four females. (C) rqRT-PCR analysis of *AaLT* abundance in midguts 0–72 h pbm from females treated with *dsEGFP* or *dsMIR* RNA. Transcript levels were standardized to 1 for the 0 h pbm samples from *dsEGFP*-treated females (negative control) while transcript abundance for the other samples is expressed relative to the negative control. (D) rqRT-PCR analysis of *AaSPVI* abundance in midguts 0–72 h pbm from females treated with *dsEGFP* or *dsMIR* RNA. Transcript levels were standardized as in C. (E) Trypsin activity in midguts 24–72 h pbm in females treated with *dsEGFP* or *dsMIR* RNA. A minimum of 5 midguts was individually assayed per treatment. Different letters above a given treatment indicates means that significantly differ (F_5, 75_ = 20.2, P<0.001) followed by comparison of all pairs of means using the Tukey-Kramer procedure, α = 0.05).

Rescue of late phase trypsin-like activity in decapitated blood-fed females by ILP3 clearly implicated the insulin signaling pathway in the regulation of blood digestion. Yet prior results [17] and our own observations also showed that decapitation delays but does not fully inhibit blood bolus darkening, which suggested that regulation of blood meal digestion involves other factors. Exploring what these factors might be and how they interact with the insulin signaling pathway was not feasible in a decapitated background, because decapitated females cannot fully mature or oviposit eggs. We therefore used an RNA interference (RNAi) approach to disable insulin signaling by injecting newly eclosed females with double stranded (ds) MIR RNA, holding them for 5 days with water, and then blood feeding on day 6. Using this regimen, we determined that dsMIR treatment reduced *AaMIR* transcript and protein abundance in the mosquito midgut compared to injection of a dsEGFP control (Fig. 2A). Consistent with prior studies [16], dsMIR treatment had no effect on transcript abundance of *AaET* at 2 h pbm (Fig. 2B). In contrast, dsMIR treatment did reduce transcript abundance of *AaLT* and *AaSPVI* at 24 h pbm relative to mosquitoes treated with dsEGFP (Fig. 2C, D). Correspondingly dsMIR treatment also reduced trypsin-like activity (Fig. 2E). However, the abundance of these transcripts and trypsin-like activity then rose at 48 h pbm in dsMIR-treated mosquitoes before falling to similar levels as controls at 72 h pbm (Fig. 2C–E). These findings mirrored our results with decapitated mosquitoes, and also showed that loss of insulin signaling delayed but did not fully inhibit late phase trypsin-like activity in the midgut.

### 2.3. *In vivo* knockdown of the MIR also delays vitellogenesis and reduces the number of oviposited eggs

**Figure 3:**
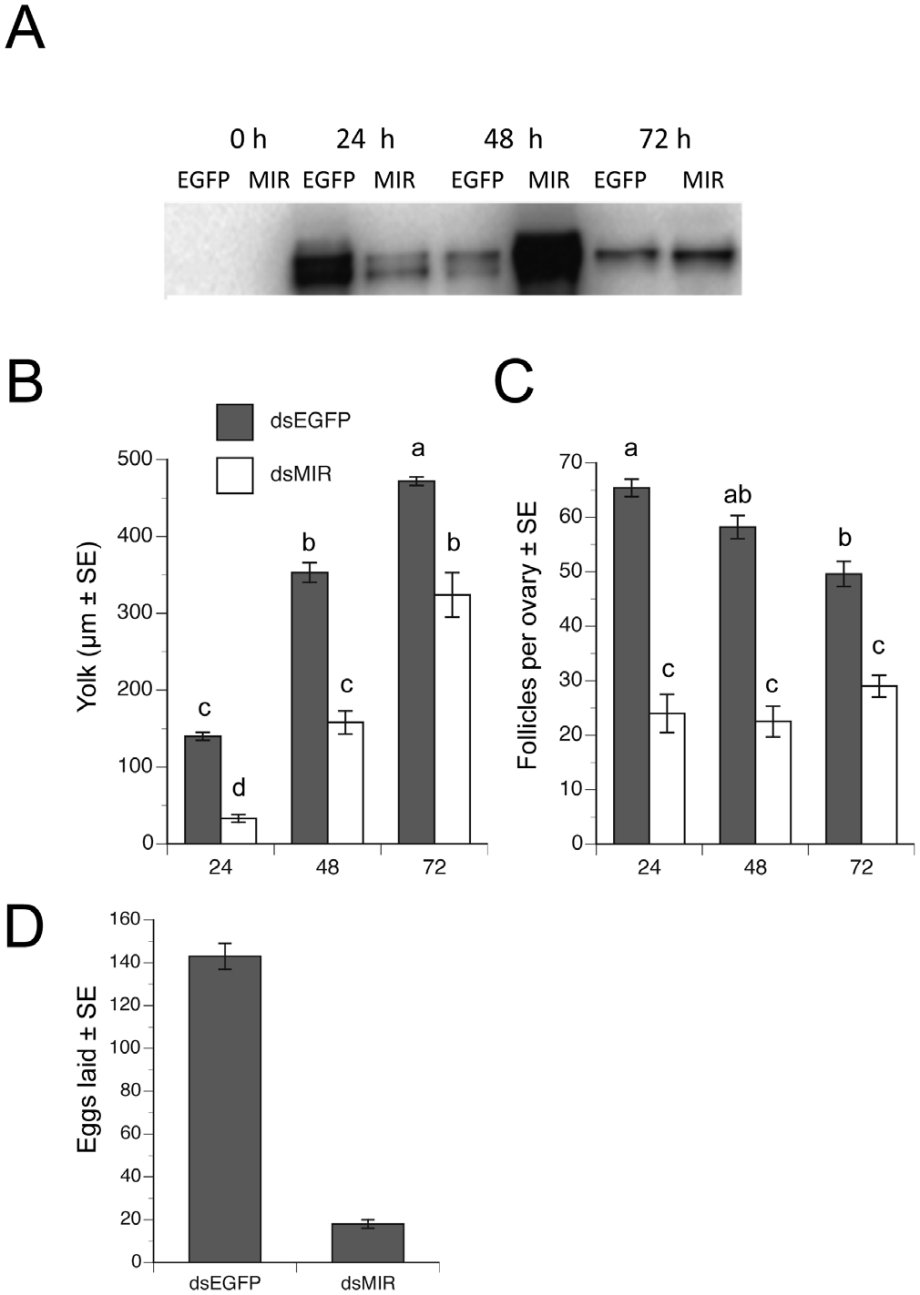
Knock down of *MIR* expression delays vitellogenin (Vg) expression and reduces egg maturation in blood-fed females. **(**A**)** Immunoblot of fat body (0.05 abdomen pelt/lane) collected 0–72 h pbm from females injected with *dsEGFP* or *dsMIR* RNA, extracted as 2 pelts, and subjected to SDS-PAGE followed by transfer to nitrocellulose and detection with an Vg antibody. **(**B**)** Yolk deposition per oocyte 24–72 h pbm in females treated with *dsEGFP* or *dsMIR* RNA. Different letters above a given bar in the graph indicate means that significantly differ (F_5, 211_ = 186.7, P<0.0001, followed by comparison of all pairs of means using the Tukey-Kramer procedure, α = 0.05). (C) Number of ovary follicles with ≥60 µm of yolk 24–72 h pbm in females treated with *dsEGFP* or *dsMIR* RNA. Different letters above a given bar in the graph indicate means that significantly differ (F_5, 195_ = 60.1, P<0.0001, followed by comparison of all pairs of means using the Tukey-Kramer procedure, α = 0.05). **(**D**)** Number of eggs laid by females treated with *dsEGFP* or *dsMIR* RNA (F_1, 61_ = 140.0, P<0.0001).

Experiments with blood-fed females indicated that dsMIR treatment also resulted in delayed expression of *Vg* by the fat body and accumulation of yolk by oocytes (Fig. 3A, B). In control dsEGFP-injected females, oocytes accumulated more than 400 µm of yolk by 72 h pbm and were ready for oviposition (Fig. 3B), but dsMIR-treated females produced significantly fewer oocytes with less yolk (Fig. 3B, C). Correspondingly, dsMIR-treated females laid an average of only 18 eggs while control females laid more than 140 (Fig. 3D).

### 2.4. dsMIR plus rapamycin treatment severely disables blood meal digestion and egg laying

As previously noted, prior studies implicate TOR signaling in the translation and release of *AaET* [9], [10], [16]. How late phase trypsin like genes are regulated is unknown, but the previous results together with evidence that insulin and TOR signaling often interact [18], [19], [20] led us to hypothesize that both ILP release from the brain and amino acid sensing through TOR are involved. If true, inhibition of TOR should reduce late phase trypsin-like activity in a manner similar to disruption of insulin signaling, whereas disruption of TOR and insulin signaling together should result in stronger suppression of digestion and egg development than either treatment alone.

**Figure 4:**
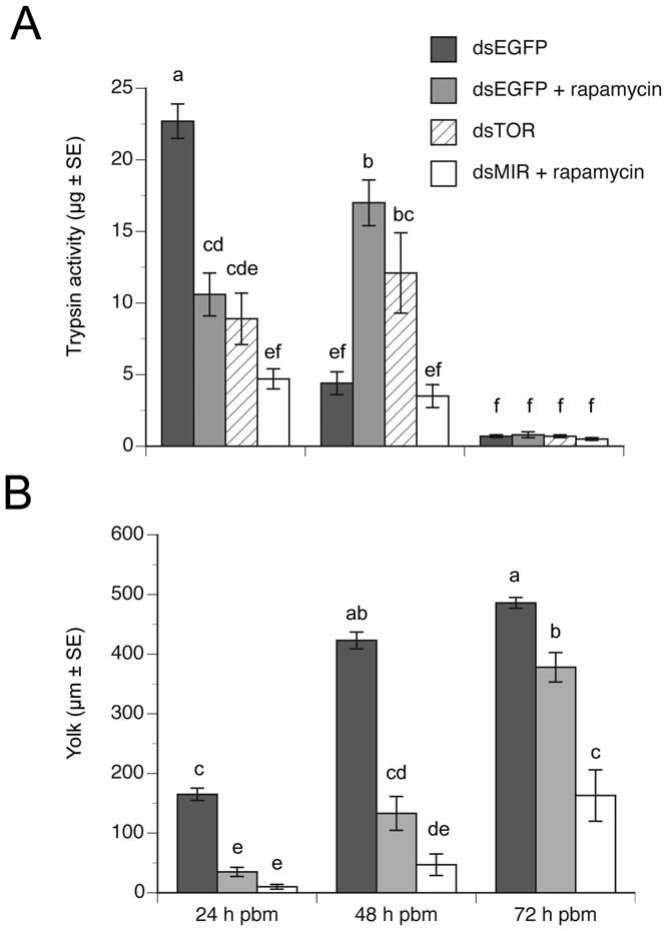
*dsMIR* RNA plus rapamycin treatment strongly reduces midgut trypsin activity and yolk deposition in blood-fed females. (A) Trypsin activity 24–72 h pbm in the midgut of females treated with *dsEGFP* RNA, *dsTOR* RNA, *dsEGFP* RNA plus rapamycin, or *dsMIR* RNA plus rapamycin. Different letters above a given treatment indicate means that significantly differ (F_10, 123_ = 35.5, P<0.0001) followed by comparison of all pairs of means using the Tukey-Kramer procedure, α = 0.05). (B) Yolk deposit per oocyte 24–72 h pbm in females treated with *dsEGFP* RNA, *dsTOR* RNA, or *dsMIR* RNA plus rapamycin. Different letters above a given bar in the graph indicate means that significantly differ (F_8, 129_ = 80.0, P<0.0001 and Tukey-Kramer procedure, α = 0.05).

We tested these predictions by injecting newly emerged females with dsMIR, dsTOR, or dsEGFP RNA (control) as described above. We then injected subsets of dsMIR and dsEGFP-treated females with 20 pmol of rapamycin the day prior to blood feeding followed by measurement of late phase trypsin-like activity and yolk deposition at 24, 48, and 72 h pbm. Females treated with dsEGFP RNA plus rapamycin exhibited a 24 h delay in late phase trypsin-like activity and a reduction in yolk deposition (Fig. 4A, B) that were very similar to the effects of treating mosquitoes with dsMIR RNA alone, (see Fig. 2E, 3B). Treatment with dsTOR RNA resulted in knockdown of *AaTOR* mRNA in the midgut (data not presented), which in turn reduced trypsin activity and yolk deposition in a manner that was also very similar to mosquitoes treated with dsEGFP RNA plus rapamycin (Fig. 4A, B). Of greatest significance though was our finding that females treated with dsMIR RNA plus rapamycin exhibited lower late phase trypsin-like activity and yolk deposition than females treated with dsMIR RNA, dsTOR RNA, or rapamycin alone (Fig. 4A, B). Moreover, no mosquitoes treated with dsMIR RNA plus rapamycin laid any eggs.

### 2.5. Amino acids enhance ILP3-induced expression of late phase trypsin-like genes in the midgut

**Figure 5:**
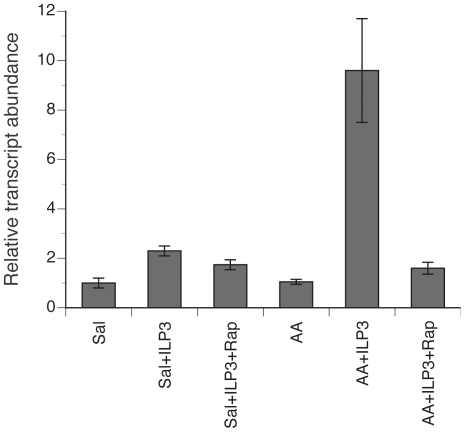
Amino acids enhance ILP3 stimulation of *AaSPVI* expression in midguts in vitro. Midguts were dissected from non-blood fed females and incubated for 3 h in saline (Sal), amino acid supplemented saline (AA), saline plus 20 pmol ILP3 (sal + ILP3), saline plus ILP3 and 100 pmol rapamycin (sal+ILP3+Rap), amino acid saline plus ILP3 (AA+ILP3) or amino acid saline plus ILP3 and rapamycin (AA+ILP3+Rap). rqRT-PCR analysis was then performed to determine relative transcript abundance of *AaSPVI*. Transcript levels were standardized to 1 for the saline-treated sample (negative control) while transcript abundance for the other treatments is expressed relative to the negative control. Each treatment was replicated three times using midguts from four females.

We fully recognized the preceding *in vivo* experiments resulted in knockdown of the MIR in more than the midgut given our own previous results showing knockdown in the ovary [3], [4] and other studies showing knockdown in the fat body [5]. We thus also recognized that the negative effect of MIR knockdown on digestion could reflect either disabled insulin signaling in the midgut or indirect interactions involving disabled insulin signaling in other organs. To address whether 1) ILP3 directly induces the expression of late phase trypsin-like genes, and 2) amino acids play a role in this response, we dissected midguts from non-blood females and placed them in either saline or amino acid supplemented saline [5]. We then cultured the midguts or added ILP3 (20 pmol) alone or with rapamycin (100 pmol) to the cultures for 3 h and then analyzed *AaSPVI* or *AaLP* expression by relative quantitative real time (rqRT)-PCR. We detected a basal level of *AaSPVI* expression in saline cultures and a two-fold increase in transcript abundance in saline cultures containing ILP3 (Fig. 5). The addition of rapamycin also had no effect on the increase observed after addition of ILP3 (Fig. 5). Midguts incubated in amino acid supplemented saline also showed a basal level of *AaSPVI* expression, but adding ILP3 induced a ten-fold increase in transcript abundance that was blocked by rapamycin (Fig. 5). The response of *AaLT* to these treatments was near identical (data not presented), while the addition of 20E (1 µg) to midguts in saline or amino acid medium had no effect on the expression of either *AaSPVI* or *AaLT* (data not presented).

These findings indicated that amino acids alone do not induce the expression of late phase trypsin-like genes, but amino acid sensing through TOR does enhance expression stimulated by ILP3. Intriguingly, these data were also opposite to prior studies with the fat body showing that neither insulin nor 20E alone induced the expression of *Vg*, but both enhanced expression stimulated by amino acid sensing through TOR [5], [12], [13]. We repeated these *in vitro* fat body experiments under our culture conditions and observed results very similar to those in the literature (data not presented). We thus concluded that the relative roles of insulin and TOR signaling in regulating blood meal digestion and *Vg* synthesis differ between the midgut and fat body.

### 2.6. Amino acids also enhance ILP3-induced production of ECD by the ovaries

**Figure 6:**
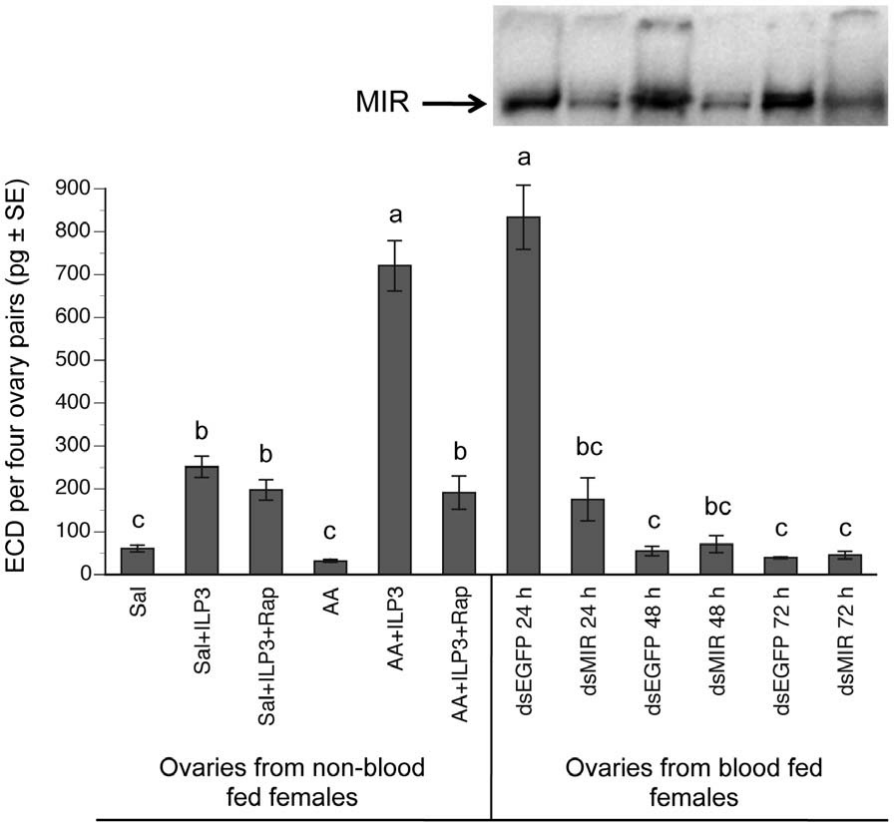
Amino acids enhance ILP3 stimulation of ovarian ECD production in vitro. The left side of the graph shows the amount of ECDs produced by ovaries (4 ovary pairs per sample) from non-blood fed females after a 6 h incubation in saline (Sal), amino acid supplemented saline (AA), saline plus 20 pmol ILP3 (sal + ILP3), saline plus ILP3 and 100 pmol rapamycin (sal+ILP3+Rap), amino acid saline plus ILP3 (AA+ILP3) or amino acid saline plus ILP3 and rapamycin (AA+ILP3+Rap). The right side of the graph shows the amount of ECDs produced by ovaries from females injected with *dsMIR* or *dsEGFP* RNA (day 1) and blood fed on day 6. Ovaries were then dissected 24, 48 and 72 h pbm and incubated for 6 h in saline. The immunoblot above the graph shows ovary extracts (2 ovaries per lane) probed with an anti-MIR antibody. Each lane on the blot corresponds to the treatment shown directly below on the graph. ECD amounts were determined by RIA using a minimum of four independent samples for each treatment. Overall, ECD amounts vary among treatments (F_11, 98_ = 81.8, P<0.0001). Different letters above a given bar indicate means that significantly differ (Tukey-Kramer procedure, α = 0.05).

Since our own prior studies showed that ILP3 directly stimulates the ovaries to produce ECD [3], we assessed whether amino acid sensing also plays a role in this response similar to our findings with the midgut. As with our in vitro midgut assays, we measured ECD production by incubating ovaries from non-blood fed females (non-oogenic) in saline or amino acid supplemented saline, followed by the addition of ILP3 or rapamycin. Our results showed that adding ILP3 to saline stimulated ECD production (∼200 pg/4 ovary pairs/6 h) to previously reported levels [3], while rapamycin had no effect on this response (Fig. 6). Ovaries incubated in amino acid supplemented saline also produced basal ECD amounts, but the addition of ILP3 to amino acid medium increased ECD production more than three fold (∼700 pg/4 ovary pairs/ 6 h) over the saline plus ILP3 treatment, while rapamycin (100 pmol) blocked this enhancement (Fig. 6).

We then examined whether these outcomes were consistent with the effects of blood feeding in mosquitoes pretreated with dsMIR or dsEGFP (control) RNA. As with our previous in vivo experiments, we injected newly emerged females with dsMIR or dsEGFP RNA followed by blood feeding on day 6. We then measured ECD amounts by dissecting ovaries at 24, 48 and 72 h pbm and incubating them for 6 h in saline. Note that 24 h pbm is when blood meal digestion and peak levels of ovarian ECD production normally occur [21], while 48 h pbm coincided with the delay in blood meal digestion that occurred after dsMIR treatment (see Fig. 2C–E). Immunoblot analysis showed that dsMIR RNA reduced but did not fully silence MIR protein levels in the ovaries relative to females treated with dsEGFP (Fig. 6). At 24 h pbm, ovaries from control mosquitoes produced large amounts of ECD (∼800 pg/4 ovary pairs/ 6 h), whereas ovaries from dsMIR RNA treated mosquitoes produced 4-fold lower levels of ECD (∼200 pg/4 ovary pairs/6 h) (Fig. 5). By 48 h pbm, ECD production by ovaries fell to basal levels in both dsMIR-treated and control mosquitoes and remained so at 72 h pbm (Fig. 6).

## Discussion

Blood feeding has long been recognized as essential for reproduction by disease-transmitting mosquitoes, yet it has remained unclear how *A. aegypti* and other mosquitoes coordinate digestion with vitellogenesis and whether this coordination is essential for normal egg production. In the first part of this study, we identify a previously unknown role for ILPs from the brain by showing that insulin and TOR signaling interact to regulate the timing of late phase trypsin like gene expression and blood meal digestion. Our *in vivo* assays show that ILP3 is able to restore digestion to normal levels in decapitated females, and our *in vivo* RNAi experiments indicate that loss of ILP signaling in the midgut delays late phase trypsin-like gene expression. In the second part of our study, we show that amino acids do not stimulate the expression of late phase trypsin-like genes in the midgut, but their presence greatly enhances the ability of ILP3 to directly stimulate late phase trypsin-like gene expression. We further show that ILP3 and amino acids have a very similar effect on ECD production in the ovaries, whereas in the fat body our results corroborate earlier studies [5], [12], [13] showing that 20E and ILPs alone are unable to stimulate *Vg* expression but their presence enhances expression induced by amino acid sensing.

Taken together, our results indicate that ILPs released from the brain after blood feeding function as central regulators of reproduction by directly and synchronously inducing expression of late phase trypsin-like enzymes by the midgut and ECD production by the ovaries. This trypsin-like activity results in the uptake and release of amino acids from the midgut into circulation where nutrient sensing by the fat body serves as the primary inducer of high level YP synthesis by the fat body. Our results further show that loss of ILP signaling severely reduces the number of mature oocytes females produce and the number of eggs they oviposit. The stark reduction in the number of eggs laid is especially striking although why delays in digestion and vitellogenesis so severely impacts oviposition is unclear. One possibility is that digestion and vitellogenesis may actually need to be tightly coordinated with yolk packaging into follicles in order for oviposition to proceed normally.

Our results clearly underscore a critical and complementary role for ILPs and TOR in regulation of blood meal digestion, ecdysteroid production, and YP biosynthesis. However, the molecular mechanisms underlying insulin and TOR signaling in mosquitoes largely remain unclear. Insulin signaling depends upon phosphorylation of Akt, which together with TOR functions as a master regulatory kinase of metabolism, growth, and other physiological processes (summarized in [20], [22]). However, the downstream signaling events through which Akt and TOR interact are complex and incompletely understood. Thus, while TOR can be activated in response to insulin and Akt can be activated by TOR, studies also show that TOR can be activated independently of insulin signaling by sensing nutrients directly [22]–[26]. TOR dependent feedback mechanisms can also impact upstream pathways that result in attenuated phosphorylation of Akt and differential responses by target cells to insulin [27]. A microRNA *miR-275* was recently implicated in regulating blood meal digestion through TOR [28]. However, much more work is needed to elucidate the relative roles of insulin and TOR signaling in activating distinctly different processes in the midgut, fat body, and ovaries that culminate in egg maturation.

Studies with *Drosophila* and other insects indicate that regulation of metabolism and growth by insulin signaling occurs in the context of an intricate communication network between tissues [18], [22], [29], [30]. Communication between neurosecretory cells in the brain that are the source of most ILPs, tissues that produce ECDs (prothoracic glands, ring gland, and ovaries), and the fat body is essential for the coordination of metabolism and growth that underpin development and reproduction. Outside of the results reported here, known effects of ILPs on the gut are limited to evidence that ablation of ILP brain cells reduced expression of an alpha 1-1-4 glucosidase (Tobi) in gut and fat body that is implicated in the regulation of glycogen stores in *Drosophila* [31]. Our results show that digestion in the gut is directly stimulated by an ILP found in the brain, thus providing the nutrients needed by tissues to complete egg maturation in female *A. aegypti*. This heretofore unrecognized role for ILPs suggests that the retardation of growth and development and extended lifespan associated with disruption of ILP release or signaling in model invertebrates may be due in part to altered digestion and associated changes in nutrient availability.

## Materials and Methods

### 4.1. Mosquitoes, animal care approval, and reagents

The UGAL strain of *A. aegypti* was used in all experiments. Females were fed on an anesthetized rat for blood feeding experiments (UGA Animal Use Protocol A2010-6-094). Protocol A2010-6-094 was reviewed and approved by The University of Georgia Institutional Animal Care and Use Committee (IACUC) who oversees and provides veterinary care for all campus animal care facilities. IACUC is accredited by the Association for Assessment and Accreditation of Laboratory Animal Care International (AAALAC), is licensed by the US Department of Agriculture, and maintains an Assurance of Compliance with the US Public Health Service. ILP3 used in the study was synthesized and purified as previously described [3], while 20-OH ecdysone (20E) was purchased from Sigma and rapamycin from LC Laboratories.

### 4.2. RNA extraction and rqRT-PCR

Total RNA was extracted from different tissues using Trizol reagent (Invitrogen). One µg of DNase-treated total RNA was used for cDNA synthesis (iScript cDNA synthesis kit, BioRad). Tissue cDNA was diluted 10 fold and used 1 µl/20 µl for RT-PCR or 1 µl/10 µl for rqRT-PCR reaction mixtures containing specific primers for *A. aegypti* (*Aa*) *Vg* (Genbank Accession Number U02548; forward 5′CAGAAGTTCGGTGCTCCTTC 3′; reverse 5′ TTCGAAGCCGAAGTCGTAGT 3′), *AaMIR* (U72939; forward 5′ CATTCCCGCTGAGCTACTTC 3′; reverse 5′ CGTCAGCACCTTCCTTCTTC 3′), *early trypsin* (*AaET*, X64362.1; forward 5′ ACCGTGGCAGATGGAGCTATG 3′; reverse 5′ GGCATAACCAGCGCAGATCAT 3′), *late trypsin* (*AaLT*, M77814.1; forward 5′ GGAAGTGATACCTTTACCGACCG 3′; reverse 5′ GATCACCAACGGGCTGTAGGC 3′), or *serine protease VI (5G1)* (*AaSPVI*, GQ398048; forward 5′ AGGAATGCCACAAGGCTTACTTGA 3′; reverse 5′ CCATAACCCCAGGATACCACT 3′). Reactions for rqRT-PCR included iQ SYBR Green Supermix (BioRad; 5 µl/tube) and were run on a Rotor Gene Real Time-PCR cycler (Corbett) for 40 or 45 cycles under the following conditions: *AaVg*, denaturation at 95° for 20 sec, annealing at 56° for 30 sec and extension at 72° for 30 sec; *AaMIR*, denaturation at 95° for 20 sec, annealing at 56° for 30 sec and extension at 72° for 30 sec; *AaET*, denaturation at 95° for 20 sec, annealing at 56° for 20 sec and extension at 72° for 30 sec; and *AaLT/AaSPVI*, denaturation at 95° for 20 sec, annealing at 58° for 30 sec and extension at 72° for 30 sec. All reactions were run in quadruplicate with control templates being 18 s ribosomal RNA [32]. Data were analyzed by the ΔΔCt method as previously outlined [33].

### 4.3. RNAi assays and immunoblotting

dsMIR and dsEGFP RNAs were produced using the T7 High Yield Transcription kit (Fermentas) [4] as was *dsTOR* RNA generated from the 3′ region of *AaTOR* using the primers *AaTOR* forward 5′-GCCGTGCTGGAAGCTTTC-3′, reverse 5′-GATTTCCGGTGTCTACCAGAAAGG-3′ and first strand cDNA from ovary RNA as template. dsRNAs were thereafter treated with DNase1 and RNaseA, precipitated, and resuspended in nanopure water at 4 µg/µl for storage at −20°C. Mosquitoes were injected with 2 µg of dsRNA at 12 to16 h post-eclosion followed by collection of specific tissues for rqRT-PCR analysis as described above. For immunoblots, midgut, abdominal wall/fat body, and ovaries were dissected at different time points in physiological saline and placed in sample buffer containing 3 parts Roche protease inhibitor cocktail (1X) and 1 part Laemelli 4X sample buffer. Samples were then run on 4–20% SDS-PAGE gels, transferred to nitrocellulose membrane (Protran 0.2 µm, Whatman), and probed with MIR (377E, 1∶6000) or *A. aegypti* Vg (R2, 1∶100,000) polyclonal antisera. Primary antibodies were then detected using a peroxidase-conjugated goat anti-rabbit secondary antibody (Sigma) and visualized with chemiluminescent substrate (Amersham ECL).

### 4.4. Bioassays

Yolk deposition was measured along the anterior-posterior axis of 10 oocytes/ovary pair of experimental females at different times pbm. Ovary follicles (egg chamber) that contained ≥60 µm of yolk were counted as mature follicles. For oviposition, at least 10 females per treatment were kept individually in small cages lined with moist paper towel. The number of eggs laid by each female was counted 96 to 120 h pbm.

Late phase trypsin-like gene expression in midguts and ECD production by ovaries were quantified by dissecting each tissue from non-blood-fed females and incubating them alone or with ILP3 or rapamycin in standard [21] or amino acid supplemented saline [5] for 3 or 6 h at 27°C (1 midgut/60 µl/dish or 2–4 ovary pairs/60 µl/dish). Trypsin-like gene expression was determined by rqRT-PCR as described above. For measurement of ECD titers, samples (50 µl) were frozen and processed for radioimmunoassay using an ECD antiserum [25].

Trypsin-like activity was measured in midguts dissected from females at different times pbm. Each midgut was transferred to 100 µl of 20 mM Tris (pH 8.0 with 20 mM CaCl_2_), sonicated, and then centrifuged (14,000 x *g* for 2 min). Midgut supernatants were frozen (−80°C), and aliquots (0.1 equivalent for early trypsin and 0.05 equivalent for late trypsin) were added to 100 µl of 4 mM Nα Benzoyl-L- Arginine-p-Nitroanilide (BAPNA) for 10 min followed by measurement of absorbance at 405 nm using a Biotek plate reader. Activity was quantified based on trypsin standards (bovine pancreas, Sigma T1426). Each treatment was replicated five times.
